# Effects of cow placenta extract on cognitive function in aged dogs: a randomized controlled trial

**DOI:** 10.1093/jvimsj/aalag125

**Published:** 2026-07-23

**Authors:** Jiang Lu, Wei Lu, Xiao-liang Qian

**Affiliations:** Department of Companion Animal, Jiangsu Agri-animal Husbandry Vocational College, Taizhou 225300, China; Department of Companion Animal, Jiangsu Agri-animal Husbandry Vocational College, Taizhou 225300, China; Department of Companion Animal, Jiangsu Agri-animal Husbandry Vocational College, Taizhou 225300, China; Canine Disease Outpatient Department, Wuxi Paideshi Pet Hospital, Wuxi 214000, China

**Keywords:** aged dogs, cognitive function, cow placenta extract, oxidative stress

## Abstract

**Background:**

Cow placenta extract (CPE) has demonstrated beneficial effects in antiaging; however, its effect on age-related cognitive decline remains unclear.

**Hypothesis/Objectives:**

To evaluate the potential therapeutic benefits of CPE in improving cognitive function in aged dogs.

**Animals:**

The aged dogs (≥7 years) with a Canine Social and Learning Behavior Survey (CSLB) score of ≥ 30 owned by clients.

**Methods:**

A 6-month randomized controlled trial. Eighty-eight dogs received CPE (*n* = 45) or placebo (*n* = 43). Canine Social and Learning Behavior Survey questionnaires were administered, and serum oxidative stress markers were assessed.

**Results:**

By month 6, the mean ± SD of CSLB was 35.1 ± 4.9 in the CPE group vs 41.1 ± 5.3 (*P* < .05; Cohen’s *d* = −1.17; 95% CI for *d*: −1.63 to −0.72) in the placebo group. The successful therapeutic response was 77.8% (95% CI, 63.2-88.0) in the CPE group vs 9.3% (95% CI, 3.1-22.3; *P* < .001) in the placebo group. The correlation between the change (Δ%) in CSLB and change in malondialdehyde (MDA) was positive (*r* = 0.77, *P* < .001), and correlations between the Δ% in CSLB and change in glutathione peroxidase (GSH-Px) and glutathione (GSH) were negative (*r* = −0.893, *P* < .001 and *r* = −0.878, *P* < .001, respectively).

**Conclusions and clinical importance:**

A six-month CPE administration improved cognitive function and oxidative stress in aged dogs, suggesting its potential for long-term management of cognitive impairment.

## Introduction

The lifespan of companion animals, particularly dogs, has increased in China. This phenomenon can be attributed to advancements in veterinary care and nutritional research.^1^^,^^2^ However, the increased lifespan coincides with more age-related diseases, including cognitive dysfunction.^3^ Cognitive aging in dogs is classified into 3 categories: successful aging, mild cognitive impairment, and cognitive dysfunction.^4^^,^^5^ Furthermore, as dogs grow older, successful aging might advance to mild cognitive impairment, and eventually to canine cognitive dysfunction (CCD). Current survey data showed a higher prevalence of CCD in dogs of older ages.^6^^,^^7^ Canine cognitive dysfunction is marked by various abnormal behaviors, such as spatial disorientation, difficulties with navigation, disturbances in circadian rhythms, reversal of sleep–wake patterns, changes in social interaction behaviors, and a decline in house-training skills.^8^^,^^9^ The current treatment strategy for cognitive dysfunction in dogs incorporates both pharmacological interventions and behavioral correction programs.^10^^,^^11^ The pharmacological interventions mainly include using drugs that enhance neuronal function and antianxiety drugs.^12^ Despite their availability, the effectiveness of these treatments tends to vary among individuals, which can have profound implications for the quality of life of both affected dogs and their owners.^13^ Given these challenges, developing novel therapeutics that can alleviate cognitive decline in senior dogs might be beneficial in reducing the clinical symptoms and prevalence of CCD.

Oxidative damage is the main contributor to aging-related diseases. As age increases, excessive production of reactive oxygen species (ROS) and reduced production of endogenous antioxidants and antioxidant enzymes aggravate oxidative damage.^14–16^ During the aging process, oxidative damage can activate neuroinflammation through various pathways, such as lipid peroxidation of cell membranes, oxidative modifications of DNA, ROS-mediated upregulation of NF-κB, and disruption of protein homeostasis, thereby inducing neuronal damage and leading to cognitive decline.^17^^,^^18^ Antioxidants reduce oxidative damage and improve cognitive function in canine models of the aging brain.^19^ Therefore, exploring interventions to enhance antioxidant capacity could lead to effective treatments to prevent brain aging and cognitive decline.

The placenta, known as “Zi he che” in Traditional Chinese Medicine, has been utilized for centuries in Southeast Asian countries to treat memory loss, agitation, and disorientation in humans.^20^ Recently, several randomized controlled trials and case studies have been conducted on the treatment of dementia using Hominis placenta pharmacopuncture.^20^^,^^21^ However, the ethical controversies surrounding the use of human tissue have restricted the clinical application of placenta-derived materials.^22^ Studies indicate that cow placenta extract (CPE) is a natural antiaging agent, primarily composed of polypeptides, which has favorable antioxidant activity.^23^ Oral administration of CPE can increase the expression of antioxidants such as superoxide dismutase (SOD), glutathione peroxidase (GSH-Px), catalase (CAT), and glutathione (GSH) in serum, reduce the level of malondialdehyde (MDA), and increase collagen content to delay skin aging induced by D-galactose in mice.^24^ However, the effect of CPE on the antioxidant capacity and age-related cognitive decline in aged dogs has not yet been investigated in veterinary medicine. Considering the similarity in active ingredients between CPE and human placental extract, it is considered important to conduct a randomized controlled trial to examine whether CPE improves cognitive ability in aged dogs. Therefore, we conducted a randomized controlled trial to evaluate the efficacy and possible mechanisms of CPE supplementation in enhancing cognitive function in aged dogs.

## Materials and methods

### Ethics statements

The guidelines employed in this study are evident in all procedures, treatments, and animal care, as stipulated in the General Rules of Welfare of Experimental Animals in China (GB/T 42011-2022). The experimental plan was submitted to the Ethics Committee of Jiangsu Agri-animal Husbandry Vocational College, China (NSF202108). This article adheres to the reporting standards proposed by PetSORT (Standards of Reporting Randomized Trials in Pets) to ensure a complete and clear presentation. In addition, written informed consent was obtained from all animal owners.

### Preparation of CPE

Fresh placentas from Holstein cows, who were in good physical health, at full-term gestation, underwent natural delivery, and had experienced between 2 and 4 parities, were sourced from a farm in Jiangsu Province, China. The placenta was immediately washed with normal saline to remove blood, mucus, and villi. It was subsequently dried and stored at −80°C for later use. The preparation process of CPE was as follows: (1) The thawed placenta was weighed at 13.6 g and placed in 20 mL of deionized water. It was then homogenized at 12 000 × *g* in a tissue homogenizer at 4°C until no residual tissue was visible. (2) The homogenate was adjusted to a total volume of 40 mL, and its pH was set to 6.5. (3) Next, 0.5 g of papain (800 U/mg, Shanghai Yuanye Bio-Technology Co, Ltd, Shanghai, China) was added to hydrolyze proteins in cow placenta, and the mixture was hydrolyzed in a water bath at 55°C for 5 h. Afterward, the mixture was heated at 90°C for 10 min to inactivate the papain. (4) The mixture was subsequently centrifuged at 9500 × *g* for 5 min to separate the supernatant. This supernatant was then filtered through a 0.22-μm microporous membrane filter, followed by evaporation, concentration, and freeze-drying to obtain a concentrated CPE. Mass spectrometry analysis revealed the identification of 128 peptides within the CPE, with the shortest peptide comprising 7 amino acids and the longest containing 25 amino acids (Table S1).^25^ (5) The CPE was encapsulated, with each capsule containing 200 mg of CPE, by Jiangsu Yabo Animal Health Products Co, Ltd (Taizhou, China). In addition, starch was used as a placebo to produce capsules that had the same appearance and weight as CPE capsules. The production adhered to the Chinese Animal Placental Peptide Consumption Standard (Q/JLSQ 0017 S-2021).

### Animals

Dogs that were over 8 years of age and weighed more than 5 kg were collected from the databases of 3 different animal clinics in the eastern region of China. We communicated with the dog owners through WeChat and requested them to fill out an online Canine Social and Learning Behavior (CSLB) questionnaire developed by Bray et al. (2022) with slight modifications.^26^ The CSLB is described in Table S2. The period for the baseline CSLB surveys was from December 1, 2023 to January 30, 2024 in this study. The CSLB score (the range from 16 to 80) were summed from each owner’s responses, with higher values indicating more severe levels of cognitive decline. Dogs with a CSLB score ≥ 30 were recruited and screened through physical examinations, CBCs, serum biochemistry, and behavioral assessments by veterinarians. The cost of veterinary examination was funded by this project. Dogs with systemic diseases and tumors were excluded from the study. Furthermore, dogs that had received relevant medications including neuronal function enhancers, anxiolytics, and nutritional supplements with antioxidant properties for the prevention and treatment of CCD in the 3 months preceding the study were also excluded.

### Sample size evaluation

The sample size estimation was based on a 5-point difference in the mean CSLB scores between the CPE and placebo groups, with a significance level of 0.05 and a statistical power of 0.9. Assuming a 1:1 ratio for the 2 groups, a total of 33 experimental subjects and 33 placebo control subjects were required. Considering a follow-up loss rate of 20%, a minimum of 42 participants was necessary in each group, resulting in a total minimum of 84 participants.

### Intervening protocol

All participating dogs were randomly assigned to either the CPE group or the placebo group in a 1:1 ratio. The randomization list was generated using random numbers produced by Excel (WPS, Kingsoft Office Software Co, Ltd, China). The investigators and dog owners remained blind to the treatment by ensuring that the appearance of the CPE capsules and the placebo was identical. Dogs weighing between 5 and 15 kg were administered 200 mg of CPE orally, while those weighing over 15 kg received 400 mg of CPE, twice daily for 6 months. The placebo group received a placebo at the same dosage, using the same method and duration. All participating dogs were required to maintain the same diet and environment as before the trial.

### Data collection

Baseline information was collected before the trial (T0), including age, sex, breed, weight, and body condition score (BCS) based on a 9-point scoring system, and the CSLB score. At the end of the second (T1), fourth (T2), and sixth (T3) months of administration, the CSLB questionnaire was collected. This questionnaire was distributed to dog owners via WeChat 2 days before the collection.

Blood samples were collected from fasted dogs at T0 and T3 to prepare serum. The levels of MDA, SOD, CAT, total antioxidant capacity (T-AOC), GSH-Px, and GSH in the serum were assessed using commercially available kits (Nanjing Jiancheng Technology Co, Ltd, China) following the manufacturer’s protocol. In addition, CBC, serum biochemical analysis, and observation of adverse events were conducted.

### Statistical analysis

Statistical analysis was conducted using SPSS software version 20.0 (IBM SPSS Statistics, San Jose, CA, USA). Normality assessment of the data set was performed using the Shapiro–Wilk test and normal probability plots. Continuous data that followed a normal distribution are presented as mean ± SD, while continuous data that did not follow a normal distribution are reported as median and range. Categorical data are expressed as population proportions. Continuous data were analyzed using either the Student’s *t-*test or the Mann–Whitney *U* test, and effect sizes were reported as Cohen’s *d*. A larger absolute value of Cohen’s *d* indicates a greater effect size. Categorical data were compared using the chi-square test to assess statistical differences.

The differences in CSLB scores between the 2 groups were compared using repeated measures analysis of variance. A paired *t-*test was employed to compare the CBC, serum biochemical analysis, and serum oxidative-related biomarkers at T3 with baseline measurements. The change rate of individual CSLB scores was calculated using the formula: Change rate (Δ%) = (CSLB score at T3 − CSLB score at T0)/CSLB score at T0 × 100. Intergroup comparisons were performed using the Mann–Whitney *U* test. In addition, a reduction of 10% or more in CSLB scores was classified as treatment success; otherwise, the outcome was categorized as treatment failure. A *P*-value of less than .05 was regarded as statistically significant.

## Result

### Animals

**Figure 1:**
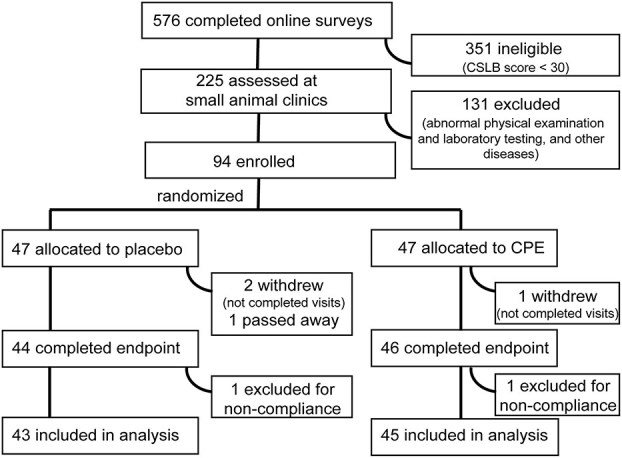
Process diagram for animal recruitment and allocation.

This study investigated a cohort of 576 senior dogs. Among these, 225 dogs with a CSLB score ≥ 30 were initially evaluated and screened, from which 131 dogs were excluded from the study due to various diseases, including diabetes, renal failure, cardiovascular disease, tumors, and other conditions. Consequently, 94 dogs were enrolled in the study. Three dogs were lost to follow-up, 1 dog died and 2 dogs were excluded due to noncompliance. Ultimately, 43 dogs of the placebo group and 45 dogs of the CPE group completed the trial (Figure 1). Table 1 presents the baseline characteristics of the placebo group and the CPE group. No statistically significant differences were observed in the baseline characteristics of the both groups, including age, fractional lifespan, body weight, BCS, sex, and CSLB score. The fractional lifespan was calculated by dividing dog’s chronological age by their expected lifespan, which was correlated with the degree of cognitive impairment in aged dogs.^28^ In addition, the breeds of dogs in both groups included Border Collies, Labrador Retrievers, Golden Retrievers, Miniature Poodles, German Shepherds, Dachshunds, Pugs, Bichon Frises, and mixed breeds, with no notable intergroup differences.

**Table 1:** The baseline characteristics of participants in the placebo group and the CPE group.

**Parameters**	**Placebo (*n* = 43)**	**CPE (*n* = 45)**	***P* value**
**Age (years)**	Mean: 10.52	Mean: 11.07	.12
	SD: 2.45	SD: 2.61	
**Fractional lifespan** ^a^	Median: 0.95	Median: 0.97	.27
	Range: 0.78-1.23	Range: 0.81-1.26	
**Sex**	Female: 8 (18.6%)	Female: 6 (13.3%)	.71
	Male: 10 (23.3%)	Male: 8 (17.8%)	
	Sterilized: 13 (30.2%	Sterilized: 14(31.1%)	
	Castrated: 12 (27.9%)	Castrated: 17(37.8%)	
**Weight (kg)**	Median: 18.7	Median: 19.2	.35
	Range: 5.2-38.5	Range: 5.7-37.2	
**BCS**	4/9: 4 (9.3%)	4/9: 4 (8.9%)	.96
	5/9: 19 (44.2%)	5/9: 18 (40.0%)	
	6/9: 10 (23.3%)	6/9: 13 (28.9%)	
	7/9: 7 (16.3%)	7/9: 6 (13.3%)	
	8/9: 3 (6.9%)	8/9: 4 (8.9%)	
**CSLB score**	Mean: 41.5	Mean: 43.2	.18
	SD: 6.3	SD: 5.7	

a Fractional lifespan was calculated by dividing dog’s chronological age by their expected lifespan. The expected lifespan was calculated using formula developed by Greer et al. (2007).^27^

Abbreviations: BCS, body condition score; CPE, cow placenta extract; CSLB, Canine Social and Learning Behavior Survey.

### The effect of CPE on CSLB score

**Figure 2:**
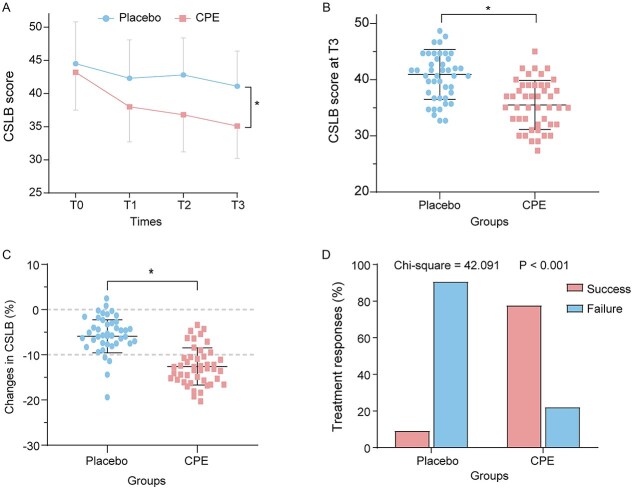
Comparison of CSLB scores between CPE and placebo groups. (A) CSBL scores at different time points. (B) CSBL scores at T3. (C) Changes of CSBL scores. Changes in individual CSLB (T3-T0) were calculated using formula: Change rate (%) = (CSLB score at T3 − CSLB score at T0)/CSLB score at T0 × 100%; (D) treatment response. Treatment response were categorized as success (CSLB score at T3 decreased by 10% or more compared to that at T0) or failure (CSLB score at T3 decreased by less than 10% or even increased compared to that at T0). ^*^P < .05. Abbreviations: CPE = cow placenta extract; CSLB = Canine Social and Learning Behavior Survey.

The mean ± SD CSLB scores in the CPE group showed a declining trend during the treatment period, decreasing from a baseline of 43.2 ± 5.7 (95% CI, 41.5-44.9) to 35.1 ± 4.9 (95% CI, 33.6-36.6, *P* < .05) at T3 (Figure 2A). The CSLB scores (35.1 ± 4.9; 95% CI, 33.6-36.6) in the CPE group were significantly lower than in the placebo group (41.1 ± 5.3; 95% CI, 39.5-42.7; *P* < .05; Cohen’s *d* = −1.17; 95% CI for *d*: −1.63 to −0.72; Figure 2B). The mean change in CSLB (Δ%) in the CPE group was −12.85 (95% CI, −13.98 to 11.72) vs −6.11 (95% CI, −7.26 to 4.96; *P* < .05; Cohen’s *d* = −1.80; 95% CI for *d*: −2.30 to 1.30; Figure 2C). A total of 35 dogs (77.8%; 95% CI, 63.2 −88.0) were considered to have a successful therapeutic response in the CPE group, whereas only 4 dogs (9.3%; 95% CI, 3.1-22.3; *P* < .001) in the placebo group (Figure 2D).

### The effect of CPE on serum oxidation-associated biomarkers

**Figure 3:**
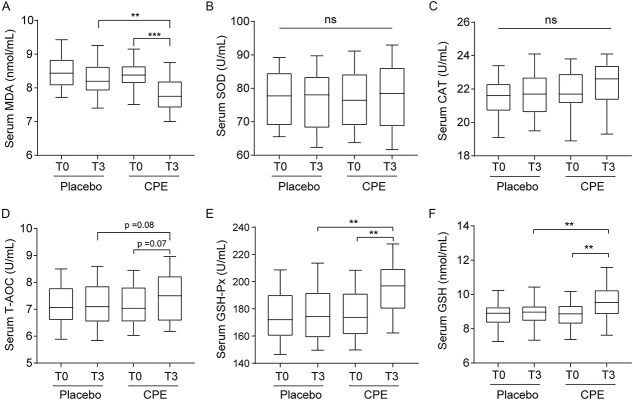
Comparison of serum oxidation-associated biomarkers between CPE and placebo groups. (A) MDA levels; (B) SOD activity; (C) CAT activity; (D) T-AOC; (E) GSH-Px activity; and (F) GSH levels. ^*^P < .05, ^**^P < .01, and ^***^P < .001. Abbreviations: CAT = catalase; CPE = cow placenta extract; MDA = malondialdehyde; GSH = glutathione; GSH-Px = glutathione peroxidase; SOD = superoxide dismutase; T-AOC = total antioxidant capacity.

Serum MDA levels in the CPE group at T3 (mean ± SD: 7.78 ± 0.67 nmol/mL) were significantly lower than both the baseline (8.37 ± 0.65 nmol/mL, *P* < .001) and those of the placebo group at the same time point (8.25 ± 0.71 nmol/mL; *P* < .01; Figure 3A). No significant differences were observed in serum SOD and CAT activities in the CPE group at T3 compared to baseline (SOD:79.6 ± 7.6 U/mL vs 77.8 ± 7.8 U/mL; CAT: 22.3 ± 2.6 U/mL vs 21.8 ± 1.9 U/mL; Figure 3B and C). Moreover, serum SOD and CAT activities showed no significant differences between the CPE group and the placebo group at T3 (SOD:79.6 ± 7.6 U/mL vs 78.3 ± 8.2 U/mL; CAT: 22.3 ± 2.6 U/mL vs 21.3 ± 2.4 U/mL). Compared to baseline, the levels of T-AOC in the CPE group did not significantly increase at T3 (7.47 ± 0.83 U/mL vs 7.18 ± 0.76 U/mL; mean difference = 0.29 U/mL; 95% CI: −0.03 to 0.61; *P* = .07). At T3, the T-AOC level in the CPE group was not significantly different from that in the placebo group (7.47 ± 0.83 U/mL vs 7.19 ± 0.79 U/mL; mean difference = 0.28 U/mL; 95% CI: −0.06 to 0.62; *P* = .08) (Figure 3D). At T3, the mean ± SD serum GSH-Px activity and GSH levels in the CPE group were significantly higher than the baseline (GSH-Px: 196.9 ± 19.1 U/mL vs 176.3 ± 18.9 U/mL, *P* < .01; GSH: 9.54 ± 0.82 nmol/mL vs 8.82 ± 0.78 nmol/mL, *P* < .01; Figure 3E and F). Furthermore, there were significant differences in serum GSH-Px activity and GSH levels between the CPE group and the placebo group (GSH-Px: 196.9 ± 19.1 U/mL vs 178.6 ± 20.2 U/mL, *P* < .05; GSH: 9.54 ± 0.82 nmol/mL vs 8.91 ± 0.81 nmol/mL, *P* < .05).

### The effect of CPE on hematologic and biochemical profiles

The CBC values in both the placebo group and the CPE group were within the normal range and supplementation of CPE for 6 months did not produce any significant effects on hematological characteristics compared to baseline (T0) and the placebo group (Table 2). There were also no significant differences in biochemical characteristics after 6 months of CPE supplementation compared to the baseline group and the placebo group (Table 3).

**Table 2:** Changes in hematologic profiles in elderly dogs with placebo and CPE supplementation.

**Parameters**	**Placebo group**		**CPE group**		***P* values**	
	**T0**	**T3**	**T0**	**T3**	***P*1** ^a^	***P*2** ^b^
**WBC (10^9^/L)**	9.87 ± 1.45	9.75 ± 1.42	9.82 ± 1.37	9.85 ± 1.44	.838	.744
**LYM (10^9^/L)**	2.42 ± 0.41	2.46 ± 0.47	2.37 ± 0.42	2.48 ± 0.41	.068	.832
**MONO (10^9^/L)**	0.47 ± 0.07	0.47 ± 0.08	0.51 ± 0.09	0.49 ± 0.06	.082	.187
**NEUT (10^9^/L)**	6.56 ± 1.11	6.43 ± 1.06	6.63 ± 1.15	6.49 ± 1.12	.384	.797
**EOS (10^9^/L)**	0.28 ± 0.05	0.29 ± 0.05	0.26 ± 0.04	0.27 ± 0.05	.128	.064
**BASO (10^9^/L)**	0.06 ± 0.02	0.06 ± 0.01	0.054 ± 0.01	0.056 ± 0.01	.162	.061
**RBC (10^12^/L)**	7.07 ± 0.30	7.02 ± 0.33	7.12 ± 0.36	7.15 ± 0.36	.503	.081
**HGB (g/dL)**	16.12 ± 0.73	16.11 ± 0.68	16.18 ± 0.75	16.22 ± 0.63	.647	.433
**HCT (%)**	46.94 ± 2.12	46.86 ± 2.15	46.99 ± 2.19	47.14 ± 2.22	.585	.550
**MCV (fL)**	67.24 ± 1.05	67.22 ± 1.09	67.25 ± 1.11	67.28 ± 1.21	.816	.808
**MCHC (g/dL)**	32.36 ± 0.32	32.38 ± 0.31	32.42 ± 0.34	32.45 ± 0.35	.485	.324
**RDWCV (%)**	15.96 ± 0.63	15.92 ± 0.64	15.95 ± 0.66	15.98 ± 0.64	.711	.661
**PLT (10^9^/L)**	245.23 ± 28.67	244.75 ± 28.53	245.86 ± 28.25	245.56 ± 27.57	.931	.893

a *P*1 is the *P*-value compared between the values at T3 and T0 in the CPE group.

b *P*2 is the *P*-value compared between the CPE group and the placebo group at T3.

Abbreviations: BASO, basophil; EOS, eosinophil; HCT, hematocrit; HGB, hemoglobin; LYM, lymphocyte; MCHC, mean corpuscular hemoglobin concentration; MCV, mean corpuscular volume; MONO, Mononuclear; NEUT, neutrophil; PLT, platelet; RBC, red blood cell; RDWCV, red blood cell distribution width coefficient of variation; WBC, white blood cell.

**Table 3:** Changes in biochemical profiles in elderly dogs with placebo and CPE supplementation.

**Parameters**	**Placebo group**		**CPE group**	
	**T0**	**T3**	**T0**	**T3**
**GLU (mmol/L)**	5.85 ± 0.53	5.88 ± 0.56	5.82 ± 0.61	5.77 ± 0.54
**TG (mmol/L)**	1.53 ± 0.25	1.51 ± 0.21	1.55 ± 0.18	1.48 ± 0.16
**CHOL (mmol/L)**	5.27 ± 0.72	5.31 ± 0.69	5.23 ± 0.78	5.19 ± 0.75
**BUN (mmol/L)**	7.82 ± 2.33	7.67 ± 2.15	7.74 ± 1.96	7.66 ± 1.92
**CREA (μmol/L)**	79.51 ± 10.26	78.83 ± 9.77	78.82 ± 11.13	78.45 ± 10.51
**TBIL (μmol/L)**	10.39 ± 2.29	10.65 ± 2.33	10.55 ± 2.45	10.36 ± 2.27
**TP (g/L)**	62.51 ± 3.75	62.86 ± 3.81	62.46 ± 3.87	63.11 ± 3.93
**ALB (g/L)**	29.26 ± 1.88	29.53 ± 1.94	29.18 ± 1.91	29.40 ± 1.98
**GLOB (g/L)**	32.77 ± 2.21	32.89 ± 2.16	32.93 ± 2.06	33.37 ± 2.15
**ALT (U/L)**	52.64 ± 6.14	51.42 ± 6.28	51.85 ± 6.54	51.33 ± 5.89
**ALP (U/L)**	107.38 ± 13.62	109.59 ± 12.75	102.94 ± 12.79	104.72 ± 12.27
**GGT (U/L)**	5.62 ± 0.43	5.54 ± 0.46	5.57 ± 0.37	5.46 ± 0.41
**AMY (U/L)**	925.45 ± 112.36	932.13 ± 114.31	928.66 ± 115.64	926.45 ± 113.06
**LIPA (U/L)**	569.92 ± 42.52	561.55 ± 42.37	564.85 ± 43.31	558.11 ± 41.54
**Ca (mmol/L)**	2.15 ± 0.22	2.17 ± 0.20	2.15 ± 0.25	2.16 ± 0.21
**PHOS (mmol/L)**	1.03 ± 0.12	1.02 ± 0.11	1.05 ± 0.10	1.05 ± 0.12

Abbreviations: ALB, albumin; ALP, alkaline phosphatase; ALT, alanine aminotransferase; AMY, amylase; BUN, Blood urea nitrogen; Ca, calcium ion; CHOL, cholesterol; CREA, creatinine; GGT, glutamyl transferase; GLOB, globulin; GLU, glucose; LIPA, lipase; PHOS, phosphorus; TBIL, total bilirubin; TG, triacylglycerol; TP, total protein.

### Correlation between the change (Δ%) in CSLB and Δ% in serum oxidation-associated biomarkers in the CPE group

**Figure 4:**
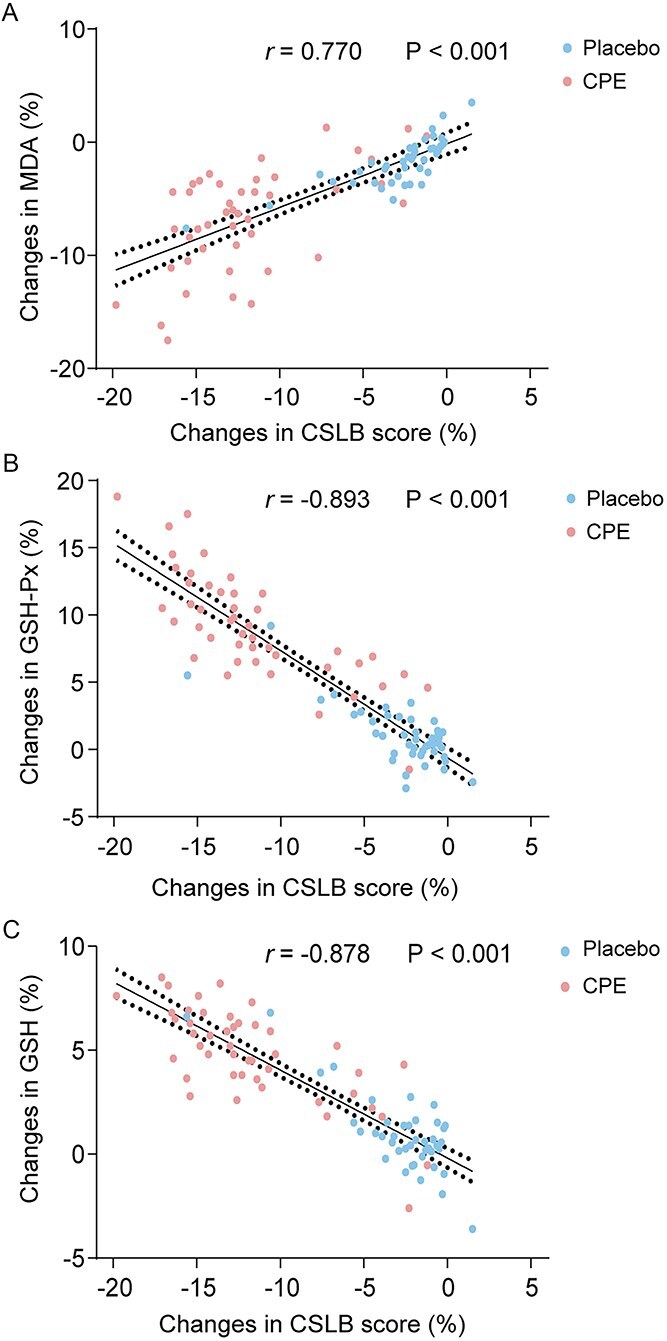
The correlation between changes in CSLB score and changes in serum oxidation-associated biomarkers in the CPE group. (A) The correlation between changes in CSLB score and changes in MDA, (B) the correlation between changes in CSLB score and changes in GSH-Px, and (C) the correlation between changes in CSLB score and changes in GSH. Abbreviations: CPE = cow placenta extract; MDA = malondialdehyde; GSH = glutathione; GSH-Px = glutathione peroxidase.

The Δ% in CSLB scores were significantly positively correlated with the Δ% in MDA (*r* = 0.77; *P* < .001), and significantly negatively correlated with Δ% in GSH-Px (*r* = −0.893; *P* < .001) and GSH (*r* = −0.878; *P* < .001), as shown in Figure 4.

### Adverse events

During the trial, 3 dogs (1 in the CPE group and 2 in the placebo group) exhibited hematuria due to bladder stones, which resolved after treatment. In addition, 1 dog in the CPE group experienced mild diarrhea, which alleviated without treatment. No other serious adverse events were observed.

## Discussion

Currently, cognitive impairment in dogs cannot be cured.^29^ In this randomized controlled clinical trial, we observed that the administration of CPE for 6 months decreased the CSLB scores in aged dogs, with no serious adverse effects attributed to the treatment. Compared to the 9.3% in the placebo group, 77.8% of the participating dogs had a successful treatment response. These findings indicate that CPE is beneficial for improving cognitive function in aged dogs.

Cognitive impairment is a condition that is often underdiagnosed. Screening questionnaires play a crucial role in assessing dogs’ behavior and cognitive function, and thus are currently the most important diagnostic tools available. Various questionnaires have been developed to evaluate the cognitive function of dogs, such as the Canine Cognitive Dysfunction Rating Scale (CCDR) and Canine Dementia Scale.^30^ The CCDR has been validated as a clinical instrument for detecting cognitive function.^31^ However, the results of the CCDR score might exhibit related subjective deviation due to the negative connotations associated with the term “cognitive dysfunction.” The content of the CSLB is largely analogous to that of the CCDR, with only minor wording modifications having been made to mitigate the negative implications of the term “cognitive dysfunction.” The effectiveness of using CSLB to assess canine cognitive function has been validated through large sample studies. A study involving a sample of 15 019 dogs participating in the Dog Aging Project revealed that a CSLB score diagnostic threshold of ≥ 50 demonstrated the highest predictive capacity for CCD. In contrast, a threshold of CSLB score > 37 exhibited a lower predictive capacity.^32^ These findings suggest that the CSLB score possesses excellent discriminating ability regarding cognitive function in dogs. In this study, the mean of CSLB score in the CPE group decreased by 12.85% compared to the baseline, which was significantly different from the 6.11% observed in the placebo group. In addition, 77.8% of the dogs in the CPE group exhibited a positive treatment response. Our results indicate that CPE has a beneficial effect on improving cognitive function in aged dogs.

As dogs grow older, their cognitive abilities naturally decline due to brain aging. The pathological mechanism of brain aging is complex. In humans, oxidative stress is one of the key mechanisms underlying brain aging and Alzheimer’s disease.^33^ Similar to observations in humans, oxidative stress in the brains of aged dogs also leads to a decline in cognitive function.^34^ The decline in cognitive function in dogs is associated with an increase in oxidative damage in the aging brain.^34^ Serum biochemical markers, such as MDA, SOD, CAT, T-AOC, GSH-Px, and GSH, are commonly important indicators for assessing oxidative damage and aging. In the present study, CPE significantly decreased serum levels of MDA and increased the activity of GSH-Px and the levels of GSH. These alterations might play a crucial role in mitigating damage induced by systemic oxidative stress, including that in the brain. Placental extract has been proven to be a natural antiaging drug, which is mainly composed of peptides with antioxidant properties. To determine whether there is a correlation between the antioxidant activity of CPE and the improvement of cognitive function, we conducted a correlation analysis between the CSLB and serum antioxidant indicators in the CPE group. The observed correlation between the improvement in CSLB scores and the decrease in MDA levels and the increase in GSH-Px and GSH, indicates that the antioxidant effects of CPE might contribute to the improvement of cognitive functions.

This study is a randomized controlled trial to evaluate the effect of placental extract on cognitive function in this species. However, several limitations exist in the present study. The relatively small sample size is a main limitation. This study recruited 88 participants, which is sufficient to detect the effects of CPE on primary outcomes such as CSLB; however, some potential positive effects might not have reached statistical significance. On the other hand, this study did not conduct subgroup analyses (eg, stratified by dog breed, sex, or baseline cognitive status) due to the small sample size. This might overlook the potential heterogeneity in responses to CPE among different subgroups. Another limitation is the short duration of the CPE intervention. Cognitive improvement is a gradual process, and a longer intervention period might be more beneficial for the cumulative effects of CPE, leading to more significant and robust cognitive enhancement. We hypothesize that prolonged and continuous supplementation might yield more lasting and stronger neuroprotective effects, such as through the sustained inhibition of neuroinflammation and promotion of neurotrophic factor expression, thereby more effectively delaying the decline in cognitive abilities. Furthermore, this study is unable to assess the safety of CPE application over the long term (eg, more than 1 year). Although no severe adverse reactions were observed in the short term (6 months), long-term effects, such as potential impacts on liver and kidney function and gut microbiota, remain unknown. Future long-term follow-up studies are needed to comprehensively evaluate its long-term efficacy and safety.

## Conclusion

In conclusion, oral CPE supplementation safely improved cognitive function and reduced oxidative stress in our study cohort. The results suggest that CPE is a new strategy to enhance the cognitive potential of dogs.

## Supplementary Material

Supplementary_material_aalag125

## Data Availability

The datasets used and/or analyzed during the current study are available from the corresponding author upon reasonable request.

## Off-label antimicrobial declaration

The authors declare no off-label use of antimicrobials.

## Institutional animal care and use committee or other approval declaration

Approved by the Ethics Committee of Jiangsu Agri-animal Husbandry Vocational College, China (NSF202108).

## Abbreviations

CAT: catalase
CPE: cow placenta extract
CSLB: Canine Social and Learning Behavior Survey
GSH: glutathione
GSH-Px: glutathione peroxidase
MDA: malondialdehyde
SOD: superoxide dismutase
T-AOC: total antioxidant capacity
