# Supplementary material for: Effects of cow placenta extract on cognitive function in aged dogs: a randomized controlled trial
Source: J Vet Intern Med. 2026 Jul 23;40(4):aalag125. doi: 10.1093/jvimsj/aalag125 (PMC13395076; doi:10.1093/jvimsj/aalag125)
Supplement: Supplementary_material_aalag125 [file supplementary_material_aalag125.zip › Supplementary Table 2.docx]

Supplementary Table 2. The CSLB used in present study

| Items | |
| --- | --- |
| Q1 | The frequency of the following behaviors exhibited by your dog: pacing back and forth, walking in circles, or wandering aimlessly without direction or purpose. |
|  | A.Never B.Once a month C.Once a week D.Once a day E.> Once a day |
| Q2 | Compared to six months ago, how does the frequency of your dog's behavior in Q1 change now? |
|  | A.Much less B.Slightly less C.The same D.Slightly more E.Much more |
| Q3 | The frequency of your dog staring blankly at walls or floors. |
|  | A.Never B.Once a month C.Once a week D.Once a day E.> Once a day |
| Q4 | Compared to six months ago, how does the frequency of your dog’s behavior in Q3 change now? |
|  | A.Much less B.Slightly less C.The same D.Slightly more E.Much more |
| Q5 | The frequency of behaviors where your dog gets stuck behind objects and is unable to navigate around. |
|  | A.Never B.Once a month C.Once a week D.Once a day E.> Once a day |
| Q6 | The frequency of behaviors that your dog collides with walls or doors. |
|  | A.Never B.Once a month C.Once a week D.Once a day E.> Once a day |
| Q7 | The frequency of behaviors that your dogs walk away or avoid being patted when you lightly tap the dog. |
|  | A.Never B.Once a month C.Once a week D.Once a day E.> Once a day |
| Q8 | The frequency of behaviors that your dog is failing to recognize familiar people or animals. |
|  | A.Never B.Once a month C.Once a week D.Once a day E.> Once a day |
| Q9 | Compared to six months ago, how does the frequency of events in Q8 change now? |
|  | A.Much less B.Slightly less C.The same D.Slightly more E.Much more |
| Q10 | The frequency of behaviors that your dog has difficulty in finding food dropped on the floor. |
|  | A.Never B.Once a month C.Once a week D.Once a day E.> Once a day |
| Q11 | Compared to six months ago, how does the frequency of events in Q10 change now? |
|  | A.Much less B.Slightly less C.The same D.Slightly more E.Much more |
| Q12 | Compared to 6 months ago, how much of time does your dog spend being active now? |
|  | A.Much more B.Slightly more C.The same D.Slightly less E.Much less |
| Q13 | Compared to six months ago, how does the frequency of behaviors that your dog urinate or defecate in an area it has previously kept clean now? (If your dog has never house-soiled, tick ‘The same’.) |
|  | A.Much less B.Slightly less C.The same D.Slightly more E.Much more |

The questions were answered using options including A, B, C, D, and E. The options were assigned to scores by a five score system (A = 1, B = 2, C = 3, D = 4, and E = 5). CSLB score is expressed as the sum of the scores of each question with weight (Q9 ×3, Q11 ×2), with the range between 16 to 80.
