# Supplementary material for: Effects of cow placenta extract on cognitive function in aged dogs: a randomized controlled trial
Source: J Vet Intern Med. 2026 Jul 23;40(4):aalag125. doi: 10.1093/jvimsj/aalag125 (PMC13395076; doi:10.1093/jvimsj/aalag125)
Supplement: Supplementary_material_aalag125 [file supplementary_material_aalag125.zip › Supplementary Table 1.docx]

Supplementary Table 1. Peptide identification list

| Sequence | Length | Missed cleavages | Mass | Charges | PEP | Score | Intensity | Intensity Papain-1 | Intensity Papain-2 | Intensity Papain-3 |
| --- | --- | --- | --- | --- | --- | --- | --- | --- | --- | --- |
| LNEQGLR | 7 | 0 | 828.44537 | 2 | 0.025035 | 122.54 | 1909800000 | 26666000 | 19286000 |  |
| TDEDTIID | 8 |  | 920.39747 | 1 | 0.038808 | 114.54 | 33473000 | 10035000 | 18678000 | 4759800 |
| HLNDDVVK | 8 | 0 | 938.48214 | 2 | 0.011373 | 118.5 | 728370000 | 125260000 |  | 82245000 |
| KGNYLILK | 8 |  | 947.5804 | 2 | 3.7424E-33 | 158.08 | 3270300000 | 914040000 | 1906400000 | 449860000 |
| ETLHDAMH | 8 |  | 952.40727 | 2 | 0.036383 | 137.44 | 599700000 | 301380000 | 239570000 | 58759000 |
| FGFDGDFY | 8 |  | 966.37595 | 1 | 0.025038 | 143.18 | 37576000 |  | 25447000 |  |
| TDDHTLIR | 8 |  | 969.48796 | 2 | 0.00002488 | 137.88 | 306710000 | 306710000 |  |  |
| RNDEELNK | 8 |  | 1016.4887 | 2 | 6.709E-46 | 163.2 | 2593600000 | 666170000 | 874590000 | 1052900000 |
| REEYLLLK | 8 |  | 1062.6073 | 2 | 7.2522E-11 | 162.5 | 4413500000 | 2346200000 | 1375400000 | 691900000 |
| GKGLVGAVT | 9 |  | 800.4756 | 2 | 0.017585 | 106.42 | 5051800000 | 5051800000 |  |  |
| VPPPFPPIQ | 9 |  | 990.55385 | 2 | 0.0053838 | 137.44 | 123170000 | 95647000 | 72406000 | 43856000 |
| FETFNVPAM | 9 |  | 1054.4794 | 2 | 0.015262 | 128.38 | 40356000 |  | 7517000 |  |
| YYAPFDGIL | 9 |  | 1057.512 | 1 | 0.029131 | 107.49 | 900480000 | 7155100 |  |  |
| FNQTDLTIK | 9 |  | 1078.5659 | 2 | 0.028495 | 104.44 | 48326000 |  | 48326000 |  |
| DNLLDDLQR | 9 | 0 | 1100.5462 | 2 | 0.00089781 | 155.38 | 636760000 |  | 77025000 | 3090400 |
| EEDIRPYIS | 9 |  | 1120.5401 | 2 | 0.016956 | 106.93 | 386120000 | 92345000 | 228610000 | 65167000 |
| FGFDGDFYR | 9 |  | 1122.4771 | 2 | 0.018901 | 119.17 | 31298000 |  | 25447000 |  |
| PQFYELLIK | 9 |  | 1149.6434 | 2 | 0.012407 | 110.81 | 1335900000 | 210040000 |  | 1125900000 |
| DEQNEEKRR | 9 | 2 | 1202.564 | 3 | 0.016611 | 73.985 | 508820000 | 141910000 | 270080000 | 113030000 |
| NWDDMEKIW | 9 |  | 1235.5281 | 2 | 2.499E-11 | 162.49 | 246690000 | 584750 | 246110000 |  |
| GPAGPIGPVG | 10 |  | 820.4443 | 1 | 2.525E-40 | 154.91 | 125110000 | 43371000 | 81737000 |  |
| GPPGTPGSPG | 10 |  | 822.38718 | 1 | 0.000035651 | 152.47 | 149310000 | 116750000 |  | 32563000 |
| YYAPFDGILG | 10 |  | 1114.5335 | 1 | 0.000021094 | 142.64 | 9494400000 | 7155100 |  |  |
| RHSQTTDDPQ | 10 |  | 1183.5218 | 2 | 4.8195E-10 | 138.98 | 139560000 |  | 12383000 | 127180000 |
| TSQEDRQLIN | 10 |  | 1202.5891 | 2 | 0.0068305 | 105.94 | 896910000 | 509100000 | 339410000 | 48400000 |
| RDNLLDDLQR | 10 |  | 1256.6473 | 2 | 0.0033132 | 112.13 | 48366000 |  | 48366000 |  |
| EEDIRPYISR | 10 |  | 1276.6412 | 2;3 | 5.7547E-15 | 142.36 | 660930000 | 165730000 | 408640000 | 86568000 |
| FTPEELEKYQ | 10 |  | 1282.6081 | 2 | 0.0058227 | 113.26 | 17089000 |  | 17089000 |  |
| DEQNEEKRRQ | 10 |  | 1330.6226 | 2 | 4.153E-10 | 148.91 | 525020000 | 141910000 | 270080000 | 113030000 |
| TNWDDMEKIW | 10 |  | 1336.5758 | 2 | 0.014798 | 117.31 | 45518000 |  | 45518000 |  |
| VEGPPGPEGPA | 11 |  | 1005.4767 | 2 | 1.2163E-06 | 125.25 | 1153000000 | 748040000 | 125690000 | 279230000 |
| GPGGLPGFYAQ | 11 |  | 1062.5134 | 2 | 0.0227 | 91.041 | 71736000 | 38100000 | 22468000 | 11168000 |
| DGLDGEDGDKG | 11 |  | 1076.4258 | 1 | 0 | 235.01 | 259010000 | 121010000 | 110570000 | 27432000 |
| AELTAPFPPVG | 11 |  | 1097.5757 | 2 | 0.026889 | 101.64 | 9185900 | 3566100 | 4431100 | 1188700 |
| NGKTSDYLLLG | 11 |  | 1179.6136 | 2 | 0.032393 | 96.492 | 671520000 | 441420000 | 190370000 | 39722000 |
| NLQTDLSDGLR | 11 |  | 1230.6204 | 2 | 0.029653 | 87.913 | 8018900 |  | 8018900 |  |
| SPGDELYKIIQ | 11 |  | 1261.6554 | 2 | 1.2535E-186 | 191.48 | 19364000 | 9997100 | 9367000 |  |
| ANNGKQWAEVF | 11 |  | 1262.6044 | 2 | 0.021975 | 98.523 | 27108000 | 9317500 | 14486000 | 3304800 |
| KVIDELDVKPE | 11 |  | 1283.6973 | 2 | 0.00006781 | 144.1 | 301920000 | 167910000 | 122150000 | 11849000 |
| LHEEQGEIILK | 11 |  | 1307.7085 | 2 | 0.023027 | 111.17 | 502510000 | 181140000 | 262090000 | 59276000 |
| HDPRDDDLNLR | 11 | 1 | 1364.6433 | 3 | 0.000025796 | 110.93 | 683100000 | 269430000 | 235230000 | 71628000 |
| SRFDQHQNKPE | 11 |  | 1384.6484 | 2 | 0.028208 | 100.72 | 132950000 | 80748000 | 52199000 |  |
| IINIFHQYSVR | 11 |  | 1388.7565 | 2 | 0.035045 | 86.624 | 18894000 | 11509000 | 7385100 |  |
| WELTDDKNQRF | 11 |  | 1450.6841 | 3 | 0.0096734 | 99.283 | 69961000 | 8069000 | 13303000 |  |
| VEGPPGPEGPAG | 12 |  | 1062.4982 | 1;2 | 0.0031192 | 139.97 | 4639600000 | 2713000000 | 1011600000 | 915000000 |
| FGTIGIGTPAQD | 12 |  | 1175.5823 | 1;2 | 2.0718E-11 | 187.99 | 13999000000 | 32531000 |  |  |
| TAELTAPFPPVG | 12 |  | 1198.6234 | 2 | 0.036519 | 94.114 | 10468000 | 4419800 | 5043400 | 1004400 |
| TVTAMDVVYALK | 12 | 0 | 1309.6952 | 2 | 0.0012443 | 110.14 | 32027000 | 16273000 | 7016700 | 2966700 |
| TDIDKDSNTDIL | 12 |  | 1348.6358 | 2 | 0.000019685 | 116.05 | 83960000 | 33432000 | 36587000 | 13941000 |
| EVEPSDTIENVK | 12 |  | 1358.6565 | 2 | 0.033141 | 92.538 | 421020000 | 138040000 | 282990000 |  |
| DDNDEDGDDLLH | 12 |  | 1371.5062 | 2 | 9.5713E-06 | 140.83 | 150050000 | 72303000 | 69879000 | 7862800 |
| HYDDSMPDPLPE | 12 |  | 1414.5711 | 2 | 0.014137 | 102.51 | 26496000 | 13663000 | 10595000 | 2237000 |
| DFGHIQYVAAYR | 12 | 0 | 1438.6993 | 3 | 6.8379E-17 | 103.91 | 33357000 | 5946100 |  |  |
| DLFENTNHTQVQ | 12 |  | 1444.6583 | 2 | 0.01212 | 103.08 | 25848000 |  | 25848000 |  |
| RDNLLDDLQRLK | 12 |  | 1497.8263 | 3 | 0.027526 | 121.69 | 16343000 |  | 16343000 |  |
| WELTDDKNQRFF | 12 |  | 1597.7525 | 3 | 0.023556 | 83.182 | 21372000 | 8069000 | 13303000 |  |
| VGEAGPEGPPGEP | 13 |  | 1191.5408 | 2 | 0.046727 | 59.931 | 395570000 | 217850000 | 136060000 | 41652000 |
| FGTIGIGTPAQDF | 13 |  | 1322.6507 | 1;2 | 5.8537E-48 | 244.16 | 13701000000 | 32531000 |  |  |
| QEASFTITVPPSA | 13 |  | 1346.6718 | 2 | 0.036004 | 61.161 | 45045000 | 45045000 |  |  |
| GPLPMEAIEKMAS | 13 |  | 1372.6731 | 2 | 0.041606 | 60.518 | 37478000 |  | 37478000 |  |
| GPPLSQSQKWLPS | 13 |  | 1423.746 | 2 | 0.041606 | 60.518 | 5094000 | 5094000 |  |  |
| KPEYDEAGPSIVH | 13 |  | 1440.6885 | 2 | 8.7327E-58 | 209.97 | 86906000 |  | 86906000 |  |
| LEVEPSDTIENVK | 13 |  | 1471.7406 | 2 | 2.5067E-07 | 112.13 | 1490100000 | 684680000 | 701570000 | 103810000 |
| DGRHDPRDDDLNL | 13 |  | 1536.6917 | 2 | 0.040717 | 90.653 | 537320000 | 260290000 | 257750000 | 19289000 |
| QIKQVEDDIQQLL | 13 |  | 1568.841 | 2 | 0.0125 | 89.886 | 17595000 | 4467700 | 13128000 |  |
| SRFDQHQNKPEIL | 13 |  | 1610.8165 | 3 | 0.053229 | 59.185 | 117350000 | 65627000 | 51723000 |  |
| KEEEDKDDEEKPK | 13 |  | 1617.737 | 3 | 0.00016402 | 98.592 | 38037000 | 38037000 |  |  |
| MPMWDEEEDEEAK | 13 |  | 1637.6225 | 2 | 0.0047371 | 97.633 | 10871000 |  | 10871000 |  |
| KKYDEELEERLVE | 13 |  | 1678.8414 | 2;3 | 1.1608E-14 | 128.54 | 131530000 |  | 131530000 |  |
| GPGGSVDSGPPPDR | 14 |  | 1293.5949 | 2 | 0.022498 | 85.731 | 25591000 | 13572000 | 12018000 |  |
| ESEIIDFFLGASLK | 14 |  | 1567.8134 | 2 | 0.000012816 | 110.66 | 3329600 |  | 3329600 |  |
| MRPDSTEIDQDTIN | 14 |  | 1633.7254 | 2 | 0.017724 | 87.363 | 22036000 | 12702000 | 9334500 |  |
| DGRHDPRDDDLNLR | 14 |  | 1692.7928 | 2 | 3.824E-25 | 160.52 | 87394000 | 55997000 | 31397000 |  |
| SQLEEKENKKFPVF | 14 |  | 1721.8988 | 2 | 4.5444E-25 | 134.49 | 24917000 |  | 24917000 |  |
| SRFDQHQNKPEILN | 14 |  | 1724.8594 | 2 | 3.5725E-64 | 155.02 | 280680000 | 125950000 | 142240000 | 12480000 |
| LEVEPSDTIENVKAK | 15 |  | 1670.8727 | 2;3 | 0.014401 | 90.614 | 358570000 | 200280000 | 128880000 | 29414000 |
| SPEELKGIFEKYAAK | 15 |  | 1708.9036 | 2;3 | 1.4655E-19 | 103.44 | 48181000 | 11492000 | 36689000 |  |
| AMGIMNSFVNDIFER | 15 | 0 | 1742.812 | 2 | 7.1583E-108 | 227.37 | 616430000 | 24567000 | 6686600 | 3484500 |
| DVDIIDHHDNTYTVK | 15 |  | 1783.8377 | 2;3 | 6.3927E-54 | 146.79 | 138730000 |  | 138730000 |  |
| ILSISADIETIGEILK | 16 |  | 1713.9764 | 2 | 3.2801E-54 | 146.36 | 15196000 | 10818000 | 4378200 |  |
| LFIGGLSFETTDESLR | 16 |  | 1783.8992 | 2 | 0.0028281 | 85.288 | 5578100 | 3602000 | 1309500 | 666660 |
| TITLEVEPSDTIENVK | 16 | 0 | 1786.92 | 2 | 7.9476E-20 | 177.83 | 600840000 | 3171800 |  | 853500 |
| LFIGGLSFETTEESLR | 16 |  | 1797.9149 | 2 | 0.00070574 | 126.1 | 5682000 | 3574700 | 1182000 | 925290 |
| ISQEEMEFFTTSSGNT | 16 |  | 1806.7618 | 2 | 0.02583 | 84.658 | 32553000 | 12095000 | 18671000 | 1787100 |
| KSPEELKGIFEKYAAK | 16 |  | 1836.9986 | 4 | 0.039011 | 80.638 | 373180000 | 206630000 | 166550000 |  |
| QESLENPMPCWLYLGD | 16 |  | 1950.8492 | 2 | 0.017825 | 85.958 | 21720000 |  |  | 21720000 |
| GPPGPEGPPGAPGPEGP | 17 |  | 1465.6838 | 2 | 0.024815 | 82.261 | 909790000 | 448070000 | 344450000 | 117270000 |
| AQLGVQAFADALLIIPK | 17 |  | 1767.0295 | 2 | 3.9779E-06 | 97.214 | 4728300 | 1753700 | 2357300 | 617360 |
| DDDIAALVVDNGSGMCK | 17 |  | 1778.7815 | 2 | 0.00023556 | 90.731 | 762700 | 762700 |  |  |
| NAPAIIFIDELDAIAPK | 17 |  | 1809.9877 | 2 | 3.1428E-06 | 122.46 | 1632200 | 1632200 |  |  |
| EEEIAALVIDNGSGMCK | 17 |  | 1834.8441 | 2 | 1.0574E-06 | 94.191 | 479760 |  | 479760 |  |
| LGFSEVELVQMVVDGVK | 17 |  | 1847.9703 | 2;3 | 4.6309E-40 | 157.52 | 116730000 | 108610000 | 5446700 | 2670300 |
| ESIQELHASDMPEYSVT | 17 |  | 1934.8568 | 2 | 1.0679E-57 | 143.5 | 360650000 | 105500000 | 255140000 |  |
| SRFDQHQNKPEILNLVK | 17 |  | 2065.1069 | 4 | 0.0071391 | 87.429 | 164020000 | 146090000 | 17930000 |  |
| ARGSDGSVGPVGPAGPIG | 18 |  | 1549.7849 | 2 | 0.0024549 | 87.216 | 117330000 | 60177000 | 57157000 |  |
| VLEYLTAEILELAGNAAR | 18 |  | 1945.052 | 3 | 1.0403E-06 | 97.235 | 1515000 |  | 1515000 |  |
| ESIQELHASDMPEYSVTN | 18 |  | 2048.8997 | 2 | 9.3311E-07 | 95.401 | 14627000 |  | 14627000 |  |
| SAAEMYGSSFDLDYDFQR | 18 |  | 2100.8735 | 2 | 0.00010453 | 89.231 | 1282300 | 1282300 |  |  |
| LNEQGLRDIANTPHELYR | 18 | 1 | 2138.0869 | 3;4 | 1.3097E-42 | 149.95 | 1186100000 | 26666000 | 19286000 |  |
| ITGLDPAGPNFEYAEAPSR | 19 |  | 2003.9589 | 2 | 6.9321E-26 | 116.84 | 4496600 | 4496600 |  |  |
| STAISLFYELSENDLNFIK | 19 |  | 2203.1049 | 2 | 4.5592E-207 | 182.1 | 2728600 | 2728600 |  |  |
| TIGGGDDSFNTFFSETGAGK | 20 |  | 2006.8858 | 2 | 1.13E-12 | 105.14 | 4390400 | 2616700 | 970810 | 802840 |
| GFGFVTYATVEEVDAAMNAR | 20 |  | 2146.9994 | 3 | 0.00080908 | 62.203 | 6126300 | 5501600 |  | 624730 |
| ISEAEGPPGGSGSQGSSSPSQ | 21 |  | 1901.8239 | 2;3 | 0.0014887 | 78.964 | 5512700000 | 1776900000 | 2964400000 | 771330000 |
| ALMLQGVDLLADAVAVTMGPK | 21 | 0 | 2112.1323 | 3 | 0.00001586 | 91.276 | 9483500 | 3945500 | 9091900 | 2917800 |
| DLYANTVLSGGTTMYPGIADR | 21 | 0 | 2214.0627 | 2;3 | 2.345E-99 | 166.29 | 100310000 | 1877600 |  | 1059200 |
| WGDAGAEYVVESTGVFTTMEK | 21 | 0 | 2276.0307 | 3 | 0.00083504 | 55.972 | 18658000 | 9637100 |  |  |
| AEEGIAAGGVMDVNTALQEVLK | 22 |  | 2214.1202 | 2;3 | 0 | 299.76 | 11269000 | 4003200 | 4682000 | 2583400 |
| INALTAASEAACLIVSVDETIK | 22 |  | 2288.1934 | 3 | 1.96E-42 | 121.74 | 124080000 | 124080000 |  |  |
| EVAAFAQFGSDLDAATQQLLSR | 22 |  | 2337.1601 | 3 | 1.0395E-64 | 131.09 | 4327700 | 965170 | 3362500 |  |
| SGETEDTFIADLVVGLCTGQIK | 22 |  | 2352.1519 | 2;3 | 0 | 275.23 | 46030000 | 46030000 |  |  |
| KPLVIIAEDVDGEALSTLVLNR | 22 |  | 2364.3264 | 3 | 2.9829E-10 | 92.098 | 3826800 |  | 2444800 | 1382000 |
| TLVLSNLSYSATEETLQEVFEK | 22 |  | 2500.2585 | 3 | 0 | 168.27 | 4766300 |  | 3998700 | 767620 |
| EFQRIARRDKKAFFSDQCKEIE | 22 |  | 2800.4079 | 3 | 0.032934 | 58.997 | 36416000 | 4343600 | 32072000 |  |
| LCYVALDFEQEMATAASSSSLEK | 23 |  | 2549.1666 | 3 | 1.8819E-138 | 111.66 | 22460000 | 13810000 | 5120700 | 3529300 |
| NDFQLIGIQDGYLSLLQDSGEVR | 23 |  | 2579.2867 | 3 | 6.7688E-89 | 136.56 | 10042000 | 1107800 | 6674600 | 2259600 |
| IITITGTQDQIQNAQYLLQNSVK | 23 |  | 2588.381 | 3 | 0.028736 | 40.369 | 1411700 |  | 862460 | 549250 |
| VETGVLKPGMVVTFAPVNVTTEVK | 24 | 1 | 2514.3767 | 3 | 4.4773E-08 | 93.34 | 81473000 | 5998900 | 2739300 | 2056200 |
| YDCGEEILITVLSAMTEEAAVAIK | 24 | 0 | 2625.2918 | 3 | 9.6523E-24 | 120.52 | 8893100 | 9142500 | 84223000 | 19314000 |
| SLQDIIAILGMDELSEEDKLTVSR | 24 |  | 2674.3735 | 3 | 0.015657 | 49.25 | 1793000 |  |  | 1793000 |
| AKPQHVTETFHPDPNWKNFQKPGT | 24 |  | 2803.383 | 3 | 0.042034 | 38.933 | 1463900 |  | 1463900 |  |
| FDENDVITCFANFESDEVELSYAK | 24 |  | 2841.2327 | 3 | 1.0252E-19 | 96.303 | 6162800 | 6162800 |  |  |
| TALLDAAGVASLLTTAEVVVTEIPK | 25 | 0 | 2481.3942 | 3 | 1.8235E-06 | 80.683 | 2611900 | 16005000 | 20937000 | 14046000 |
| ISQEEAGAGAGEQTCAPGSRPPDMR | 25 |  | 2571.1442 | 3 | 0.0046029 | 60.272 | 80553000 | 32044000 | 48508000 |  |
| QPRWSSSSLDGNGCTDTTMCPAYAT | 25 |  | 2762.1371 | 3 | 0.027263 | 38.237 | 198140000 | 91809000 | 82934000 | 23397000 |
| ALQEASEAYLVGLFEDTNLCAIHAK | 25 |  | 2762.3585 | 3 | 0.010882 | 41.208 | 1010400 | 1010400 |  |  |
| MTQSLLENEALNLKNQSLARQTDPS | 25 |  | 2800.4025 | 3 | 0.010522 | 37.228 | 15938000 |  |  | 15938000 |
| LSEQEMSSAREEACVRIQQALTMFP | 25 |  | 2910.3674 | 3 | 0.011725 | 36.256 | 30283000 | 30283000 |  |  |
